# Human lung on a chip: innovative approach for understanding disease processes and effective drug testing

**DOI:** 10.3389/fphar.2012.00205

**Published:** 2013-01-17

**Authors:** Jutaro Fukumoto, Narasaiah Kolliputi

**Affiliations:** Division of Allergy and Immunology, Department of Internal Medicine, Morsani College of Medicine, University of South FloridaTampa, FL, USA

**A commentary on**

**A human disease model of drug toxicity-induced pulmonary edema in a lung-on-a-chip microdevice**

by Dongeun Huh et al. (2012). Sci. Transl. Med. 4, 159ra147.

Acute lung injury (ALI) is a severe respiratory condition (high incidence and mortality rates) caused by various etiologies such as sepsis, shock, pneumonia, heart failure, aspiration, and toxic gas. Currently, there are no established therapeutic strategies to reverse ALI disease progression. In addition, the very high-concentration oxygen therapy widely used to counteract ALI-induced hypoxiemia could deteriorate the ALI (Kolliputi and Waxman, 2009). In terms of economic impact, ALI represents a significant cost, estimated at 3.5–6 billion dollars annually in the U.S. (Raghavendran et al., 2011). One of the most outstanding hallmarks of lung pathology in ALI is pulmonary edema. It is preceded by high vascular pressure and/or increased alveolar-capillary permeability, which cause the influx of vascular leakage into the alveolar space that ultimately leads to pulmonary edema. Additionally, sustained pulmonary edema is also a major symptom of ALI (Tamarapu Parthasarathy et al., 2012). Hospitalization and mortality rates associated with pulmonary edema are significantly high. The development of new drugs toward pulmonary edema and ALI is at present hindered by the poor predictive power of existing preclinical animal models. Besides that, currently available *in vitro* experimental systems for investigating pulmonary edema do not necessarily reflect the structurally and functionally intricate alveolar-capillary system, but they consist of either epithelial cell or vascular endothelial monolayer (Mehta and Malik, 2006; Kolliputi et al., 2010, 2012). One approach to meeting this challenge is the development of innovative and cost effective models to test drugs and study multifaceted human physiology in an organ-specific context. More importantly, this approach should offer the potential to design specific *in vitro* human disease models that could revolutionize drug development.

In the November 2012 issue of Sci. Transl. Med., Huh et al. presented a state-of-the-art experimental technique to mimic the intricate vascular-alveolar structure of the lung with a microchip lined with living cells that reproduces many of the features of a human lung (Huh et al., 2012). The device framework consists of four elastic polymer channels. Two channels are separated by a thin, porous, flexible membrane lined on one side with human lung epithelial cells exposed to air and on the other side with human pulmonary vascular endothelial cells that are bathed in constantly flowing culture media to mimic vascular flow (Huh et al., 2010). The two vertically apposed channels are flanked at both sides by two hollow channels, through which cyclic vacuum can be applied to recreate breathing-induced shear stress. Huh et al. first perfused the microvascular channel with a clinically relevant dose of interleukin-2 (IL-2), an anti-cancer drug that can cause pulmonary edema (Atkins et al., 1999; Briasoulis and Pavlidis, 2001). Consistent with *in vivo* IL-2-induced pulmonary edema, the human lung-on-a-chip system successfully showed vascular leakage and flow into the epithelial-sided air channel in response to IL-2 treatment regardless of added mechanical stress. Further, Huh et al. demonstrated that immune cells are not necessary for IL-2-induced pulmonary edema and that mechanical stretch, which alone had been shown to have no detrimental effect on “lung” permeability in the microdevice, greatly exacerbates IL-2-induced human pulmonary edema. These findings definitely cast new insights into the mechanism of pulmonary edema. And, importantly, those new findings could not be readily examined using other currently available methods.

Next, the authors checked if the IL-2-induced pulmonary edema-on-a-chip model is applicable to testing pharmacological agents. Using the microchip, they first tested angiopoietin-1 (Ang-1), a known endothelial junction stabilizer (Peters, 1998). Consistent with the previous reports, Ang-1 co-administration with IL-2 completely restored IL-2-induced loss of integrity in vascular-epithelial barrier function. Additionally, Huh et al. demonstrated that a new transient receptor potential vanilloid 4 (TRPV4) ion channel inhibitor, GSK2193874 (GlaxoSmithKline) completely blocked IL-2-induced increase in vascular-epithelial permeability (Thodeti et al., 2009). They also confirmed the physiological relevance of these *in vitro* results by duplicating IL-2-induced pulmonary edema using an *ex vivo* mouse ventilation-perfusion model.

This sophisticated microengineered lung system is an epoch-making invention that will facilitate better predictions on how drugs will work in people, eventually reducing the cost of drug development by avoiding unnecessary animal experiments or clinical trials. Also, it will certainly add valuable data on disease mechanisms of the lung to complement routine cell culture and animal experiments.

However, there are limitations to Huh et al.'s microdevice: (1) The lung epithelial cells used in the system are cancer cells not primary cells, which raises the question that the system might not closely recapitulate the real pulmonary function; (2) The system lacks interstitial fibroblasts and alveolar macrophages, cells involved in maintenance of lung homeostasis (Griffin et al., 1993; Shannon et al., 2001; Herold et al., 2011); (3) The microchip-based experiment, which requires well-equipped laboratory and an experienced technician, would not be easily reproduced; (4) In this report authors have not yet established the chip capability to mimic gas exchange between the air sac and bloodstream, a key function of the lung, though this chip may also be applied to lung gas exchange model. It is certainly unlikely that animal experiments would totally be replaced by the chip. However, the microdevice will undoubtedly facilitate both a better understanding of lung diseases at the molecular level and rapid testing of marketable drugs. Using the lung-on-a-chip microdevice would definitely allow effective screening of an enormous number of drugs and provide sufficient time to focus on promising candidates. The cost reduction in drug development would be followed by reduced health care costs nationally and individually. Quick drug development will be a blessing for those who are struggling with refractory diseases. In the near future, a microengineered chip equipped with key organ functions could serve as valuable resource in the pre-clinical evaluation of drugs.
